# Eustachian tube function in adults with intact tympanic membrane

**DOI:** 10.1590/S1808-86942010000300012

**Published:** 2015-10-20

**Authors:** Renata Rumy Makibara, Jackeline Yumi Fukunaga, Daniela Gil

**Affiliations:** 1 Bachelor in Speech and Hearing Therapy – Federal University of São Paulo – UNIFESP, Graduate Student in Neurogeriatrics in Speech and Hearing Therapy – University of São Paulo Hospital; 2 Bachelor in Speech and Hearing Therapy – Federal University of São Paulo – UNIFESP, Speech and Hearing Therapist – Specialized in Clinical Audiology – São Paulo Holy House of Mercy Hospital; 3 PhD in Human Communication Disorders – UNIFESP/EPM, Adjunct Professor – Department of Speech and Hearing Therapy – UNIFESP

**Keywords:** valsalva maneuver, tympanic membrane, eustachian tube

## Abstract

The Eustachian tube has the function of equilibrating the environmental pressure with inner pressure, protecting the middle ear from abrupt pressure changes.

**Aim:** To compare the Eustachian tube function in adults with and without history of otitis media and/or respiratory tract inflammation without tympanic membrane perforation.

**Materials and Methods**: The Eustachian tube function was evaluated in forty-two females and males of 18 to 55 years of age with intact tympanic membrane, tympanometric A curves and without historic of otological surgery. The patients (study group with past otological disease) were matched by a control group. The data was analyzed to compare the Eustachian tube function in both groups.

**Results:** In the study group, 21.4% of the persons presented dysfunction of the tube against 0% in the control group. There was no statistically significant difference considering the affected ear. We observed a higher percentage of men (90.9%) with normal functioning Eustachian tube in relation to women (65%).

**Conclusion:** Middle ear infections, rhinitis and/or sinusitis influenced the Eustachian tube functioning. Therefore it is important to test its function in such individuals. Other studies are important to establish a standard of normality in each condition of the test and for a better understanding of these people's complaints.

## INTRODUCTION

The auditory tube is a closed organic tube which connects the rhinopharynx and the middle ear. It is made up of a shorter bony part, 12 millimeters long, corresponding to the auditory tube semicanal; and a 24mm cartilage, which base is inserted under the rhinopharynx lateral wall mucosa^1^.

The junction between the bony and the cartilage portions form the auditory tube's isthmus which, similarly to a valve, controls air intake1. The auditory tube is closed during rest and, by the action of the soft palate tensor muscle and the soft palate elevator muscle which are activated through swallowing and/or yawning, air moves from the nasal pharynx to the middle ear^2^,^3^. This allows pressure equalization between the external air and the air pressure inside the tympanic cavity and, also, middle ear aeration. This mechanism protects the ear from rapid pressure changes, keeps the mucosa preserved and allows the tympano-ossicular unit to vibrate without interferences^2^,^3^.

The auditory tube is also responsible for draining the middle ear, protecting it from secretion build up. The soft palate tensor muscle is in charge of expelling middle ear secretion to the nasal pharynx^2^,^4^. Moreover, the secretory and hair cells present in the middle ear and in the auditory tube, are part of a system of mucociliary transport^3^.

The auditory tube function deficit is one of the major causes of otitis media (OM). With auditory tube occlusion, the middle ear ventilation is poor and insufficient, increasing the absorption of nitrogen by the middle ear, continuously generating negative pressure, resulting in OM^5^. Moreover, otitis media may be caused by constant upper airway infections and allergies (allergic rhinitis) by allergic reactions of the nasal mucosa, causing auditory tube dysfunction, or even loss in mucociliary transport function^6^.

Tympanometry is based on the acoustic immittance measures produced by positive and negative air pressure variation in the external acoustic meatus, leading to changes in physical properties in the middle ear and tympanic membrane. Immittance tests can also be used to study the ventilatory function of the Eustachian tube^7^.

Today, there are very few studies comparing the auditory tube function in individuals with an otologic past from those without a past of otitis or upper airway infection. The assessment of this audiology function is important in order to complete the assessment procedure and to check whether or not it recovers its normal function after middle ear and/or upper air infections and for better responding to the complaints of patients, often times with complaints of ear fullness and autophonia, symptoms which can be associated with tube dysfunction, conventional tympanometric measures do not reveal alterations.

Thus, the objective of the present paper is to compare the auditory tube function in adult individuals with and without a past of otitis media and/or upper airway infections with an intact tympanic membrane.

## MATERIALS AND METHODS

This is a prospective cross-sectional study, analyzed and approved by the Ethics in Research Committee of the institution under protocol #: 1043/07.

The sample was made up of 42 individuals, 22 males and 20 females. Requirements for participation were: age between 18 and 55 years, intact tympanic membrane, type A tympanometric curve, normal hearing thresholds (up to 25dBHL between 250 and 8,000Hz) and without a past of otologic surgeries.

The patients were broken down in two groups, namely: Control group (without an otological past, that is, without a past of recurrent otitis media, adenoid obstruction or upper airway allergies and without signs and symptoms of respiratory tract infection) and the Study Group (with a positive otologic past, in other words, with a past of otitis media, adenoid-caused obstruction and/ or airway allergies). Such data on the otologic past was obtained from the information reported by the patients during clinical interview. Nonetheless, they all reported having gone through prior otorhinolaryngological evaluation and diagnosis. The groups were paired as to gender and age. Those individuals with conductive hearing loss were taken off the study.

The participants were assessed at the Clinical Audiology Ward, between September of 2007 and May of 2008. All the study volunteers were recruited by the investigators by means of a personal contact. They all signed an informed consent form.

They were submitted to: visual inspection of the external acoustic meatus; anamnesis – involving issues regarding audiologic past (hearing loss, noise exposure), otologic history (ear and upper airway infection, dizziness and tinnitus) and medical history (virus infections, medications used); threshold tonal audiometry (TA); vocal audiometry (VA); acoustic immittance measures (AIM); and auditory tube function evaluation, in both ears.

In order to study auditory tube function, the following tympanograms were traced:

1. Conventional tympanogram.
2. Following that, we asked the patient to drink a glass of water in order to reduce middle ear pressure. At this time we carried out another tympanometric recording.
3. And finally, the patient was instructed to close his mouth, occlude the nostrils with two fingers and blow the air to the nose, in order to create positive pressure in the middle ear. We then did a final tympanometric register (Valsalva maneuver).
  The auditory tube function was analyzed according to the following parameters:
  • Normal tube functioning: tympanometric registers with different pressure peaks.
  • Altered auditory tube: tympanometric registers with overlapping pressure peaks.

Normal auditory tube functioning means that the tube can open actively through the contraction of the soft palate tensor muscle, being capable of withstanding negative pressures in the nasopharynx.

The altered auditory tube indicates that the patient did not effective operate the auditory tube function in changing middle ear pressure. This means that the auditory tube is temporarily or permanently out of order.

Acoustic immittance measures as well as tube function studies were carried out with the Interacoustics AT-235 machine.

The statistical analysis in this study was carried out by means of non-parametric tests, since the conditions (assumptions) for the use of parametric tests and techniques, such as normality (Anderson-Darling test) and homoscedasticity (variance homogeneity, Levene test), were not found in this data set. For the analysis we considered the values from both ears, since we noticed that by working with both values we gained reliability, since the sample doubles in size.

For the analysis of variables, described below, we tested the Equality of Two Proportions:

– Anamnesis, related to auditory and/or otologic complaints, such as tinnitus and a feeling of fullness in the ear, positive otologic past x negative otologic past within the group and between the groups;
– Characterizing the otologic past of the study group;
– Comparing auditory tube functioning between the two groups;
– Comparing the two sides in terms of auditory tube functioning;
– Comparing the auditory tube functioning between the genders;

In order to analyze pressure variation in the tube function between the groups, considering ear side, we used the Wilcoxon test because the data have pairing characteristics.

In order to analyze pressure variation in the tube functioning between the groups we used the Mann-Whitney test.

Results with statistical significance are highlighted with the (*) symbol, with a p-value of 0.05. Values tending towards statistical significance are highlighted with the (#) symbol. The first quartile (Q1) shows the distribution up to 25% of the sample and the 3rd quartile (Q3) shows distribution up to 75% of the sample. The confidence interval (CI) shows the mean variation according to a statistical probability and in this study they were built with a 95% confidence interval.

## RESULTS

First of all we compared the auditory complaint (tinnitus) in and between the groups with the negative (control) and positive (study) otologic past.

In the intergroup and intragroup analyses regarding tinnitus, we noticed that both groups had it in equal proportions (28.6%).

As far as the sensation of full ear goes, only one individual from the control group had such complaint, compared to 52.4% of the study group individuals, and the difference was statistically significant.

Table 1 depicts the otologic past characterization in the study group:

**Table 1:** Otological past distribution in the study group

History	Number.	%
Rhinitis	8	38.1%
Rhinitis, sinusitis and otitis	5	23.8%
Otitis	2	9.5%
Rhinitis and otitis	2	9.5%
Rhinitis and sinusitis	2	9.5%
Sinusitis	2	9.5%

We will continue presenting the results comparing the two sides regarding pressure values in the tube function test in both groups, being (Tables 2 and 3):

– Pressure 1: Conventional tympanogram
– Pressure 2: After swallowing water. A negative value is expected for pressure 1.
– Pressure 3: after occluding the nostrils and blow air to the nose (Valsalva maneuver). A positive value is expected for pressure 2.

**Table 2:** Comparing ear sides as to auditory tube pressure variation in the control group

control	Pressure 1		Pressure 2		Pressure 3	
	Right	Left	Right	Left	Right	Left
Mean	-15,67	-9,71	-26,05	-23,90	48,33	41,81
Median	-17	-13	-26	-25	12	6
Standard deviation	15,81	20,23	9,65	17,67	73,89	79,32
Q1	-24	-17	-30	-32	-13	-13
Q3	-13	-3	-22	-22	103	100
N	21	21	21	21	21	21
CI	6,76	8,65	4,13	7,56	31,60	33,93
p-value	0,023*		0,204		0,972	

Right.: pressure in the right ear Left.: pressure in the left ear Median: 50% of the individuals are above the median value and 50% are below it q1: distribution up to 25% of the sample q3: distribution up to 75% of the sample N: number of ears CI: Confidence interval

**Table 3:** Comparing ear side to auditory tube pressure variation in the study group

Study	Pressure 1		Pressure 2		Pressure 3	
	Right	Left	Right	Left	Right	Left
Mean	-14,57	-16,05	-27,29	-27,19	21,86	23,33
Median	-17	-17	-26	-29	0	-15
Standard deviation	20,68	14,23	15,70	14,49	61,27	73,65
Q1	-24	-24	-33	-35	-25	-25
Q3	-13	-13	-20	-22	60	28
N	21	21	21	21	21	21
CI	8,84	6,09	6,72	6,20	26,21	31,50
p-value	0,432	0,983	0,985			

Right.: pressure in the right ear Left.: pressure in the left ear Median: 50% of the individuals are above the median value and 50% are below it q1: distribution up to 25% of the sample q3: distribution up to 75% of the sample N: number of ears CI: Confidence interval

Although we did find significant differences between the two sides for pressure 1 considering the control group, we also noticed that the difference happens because of one single individual; and moreover, the difference is small and in only one of the six comparisons.

Comparing the auditory tube functioning between the groups, only the study group showed alterations in its functioning (21.4% of the individuals), and such difference was statistically significant.

Table 4 shows a comparison between both sides regarding auditory tube function in both groups. There was no significant difference between the sides in the study and in the control groups, regarding auditory tube dysfunction and normal functioning.

**Table 4:** Comparing ear sides regarding auditory tube functioning within a group.

Auditory tube functioning	Right		Left		p-value	
	Quantity.	%	Quantity.	%		
Control	Normal	21	100%	21	100%	1,000
Study	Altered	5	23,8%	4	19,0%	0,707
	Normal	76,2%	16	17	81,0%	

Considering the working of the auditory tube within the control group according to gender, we noticed that women had higher rates of tube dysfunction (35%) then men (9%), and such difference was statistically significant.

And finally, Table 5 shows the comparison between the control and study groups regarding auditory tube pressure.

**Table 5:** Descriptive vales of the auditory tube pressure variation in the study and control groups.

Auditory tube	Pressure 1		Pressure 2		Pressure 3	
	Control	Study	Control	Study	Control	Study
Mean	-12,69	-15,31	-24,98	-27,24	45,07	22,60
Median	-16	-17	-26	-28	9	-8
Standard deviation	18,19	17,55	14,10	14,93	75,79	66,92
Q1	-22	-24	-32	-35	-13	-25
Q3	-9	-13	-22	-21	103	34
N	42	42	42	42	42	42
CI	5,50	5,31	4,27	4,51	22,92	20,24
p-value	0,317		0,490		0,071#	

control.: control group. study.: study group. Median: 50% of the individuals are above the median value and 50% are below it. q1: distribution up to 25% of the sample q3: distribution up to 75% of the sample N: number of ears CI: Confidence interval

## DISCUSSION

As to the hearing complaint (tinnitus), 28.6% reported them present in both groups. Tinnitus happened because of a lesion and or a dysfunction in the sensorineural auditory system8. Usually, the hearing loss found in tonal threshold audiometry is detectable, although it can be normal having seen that a loss of up to 30% of the cochlear hair cells do not cause audiometric changes. Among many tinnitus-inducing factors, such as presbycusis, noise exposure, ototoxicity, high blood pressure; a misalignment of the mandible condyle can block the auditory tube and cause symptoms such as otalgia, dizziness and tinnitus. Thus, correlating literature findings, tinnitus did not seem to influence auditory tube functioning in these groups of individuals, having seen that both groups equally complained of it.

As far as ear fullness goes, there was a statistically significant difference between the groups, and 52.4% of the individuals in the study group reported this sensation, compared to 4.8% in the control group. In their study, Ryding et al.^9^, reported that 74% of the individuals with past of otitis media presented some ear discomfort (ear fullness). As it was also described by Sàenz et al.5and Skoner^10^ regarding individuals with allergic rhinitis or otitis, most of them had otological complaints.

Table 1 characterizes the otological past distribution in the study group. We can notice that most of the individuals (38.1%) had only rhinitis, followed by rhinitis and sinusitis and a past of otitis in 23.8% of the cases. We must stress that the information on the otological past was obtained through anamnesis, in other words, the individuals were not submitted to otorhinolaryngological evaluation in order to confirm the complaints at the time of the auditory tube evaluation, but as mentioned, they had all been diagnosed by ENT physicians.

Based on the standard deviation results, in Tables 2 and 3 we can see that pressures 1, 2 and 3 had great variability, characterizing an asymmetrical distribution, in other words, there was no pressure variation pattern, nor any homogeneity between its values considering the individuals from this study, thus corroborating the findings from Bunne et al.11, which revealed considerable pressure individual variability during auditory tube opening and closure. Moreover, we could notice that variations in pressures 1, 2 and 3 in the control group were higher in relation to the study group.

We also noticed that in the study group, the differences found in ear sides were not considered statistically significant. Now, for the control group, we noticed that in Pressure 1 there is a mild difference between ear sides - considered significant. It is important to stress that this difference exists because of one individual only and, moreover, the difference is small and it happened in only one of the six comparisons.

We noticed that the control group had a higher normal auditory tube functioning percentage (100%) when compared to the study group (78.6%), with significant differences between them. In the study group, it was not possible to equalize the negative pressure in 9 of the 40 ears or, even, the negative and positive ones in the middle ear. In the study carried out by Sàenz et al.^5^, 15.5% of the individuals with allergic rhinitis (adults and children) had some auditory tube dysfunction. Notwithstanding, higher rates of tube dysfunction were found in the studies by Ryding et al.^9^ and Skoner et al.^10^. Fifty percent of adults with upper airway allergies showed dysfunction because of dust build up in their homes^10^. Now, 57% of the patients with a past of secretory otitis media had poor auditory tube functioning, being unable to balance positive and negative pressure changes^9^. Our results, compared to the ones in the literature, showed that patients with a past of upper airway allergies or otitis media can have auditory tube dysfunctions when compared to those individuals without these disorders. Thus, it is paramount to test the auditory tube function in these individuals.

On Table 4 we notice that there were no significant differences between the ear side affected in the control and study groups. Thus, tube dysfunction can happen similarly in either the right or the left side as per described by Bunne et al.^11^ and Bylander^12^.

In the study group, there was a statistically significant difference in gender distribution, when we noticed a higher percentage of men (90.9%) with normal auditory tube function in comparison to women (65%). Ryding et al.^9^ found differences between the genders, however not significant as we found, and women had higher numbers of tube dysfunction (79% of the ears) in comparison to men (59% of the ears). Nonetheless, they did not justify such findings. Bunne et al.11 and Bylander^12^, reported that the tube dysfunction affected both genders in similar proportions.

And finally, on Table 5 we compared the auditory tube pressure variation between the control and study groups and we noticed that in pressures 1 and 2, the study group had higher negative values in relation to the control group, however without statistically significant difference. We notice that, regarding pressure 3, the study group pressure was clearly lower when compared to the control group. Nonetheless, these values indicate only a tendency towards statistical significance. We noticed that the mean value for the study group could withstand the negative and positive pressure variation in the middle ear, but these were less efficient then the control group. In their study, Saènz et al.^5^ reported that individuals with allergic rhinitis had higher negative pressure peaks found in their tympanometry, with a statistically significant difference, when compared to those individuals without this disorder. According to Bunne et al.^11^, the Valsalva maneuver was more effective in the group with normal and healthy ears when compared to the group of ears after otitis media – having a statistically significant difference, corroborating our findings.

Notwithstanding, we did not find in the literature studies comparing pressure peaks in three difference conditions of opening and closing the auditory tube, as in this study. We observed great pressure variability in the three test conditions and between the two sides for both groups. We can then conclude that the auditory tube functioned properly during opening and closing, but we lack normality in pressure values, in other words, which variation amplitude would be accepted in the three tympanogram curves and could be obtained from a study with more subjects without a positive otological past.

Thus, it would be important to have studies to establish normality values for the variation in each test condition for clinical practice and for better understanding the complaints from this group of individuals.

## CONCLUSION

Based on the critical analysis of these results of auditory tube assessment in individuals with negative and positive otological past, we can conclude that:

• Past middle ear infections, rhinitis and/or sinusitis influenced auditory tube functioning in the adult individuals analyzed.
• The study group presented higher pressure peak negative values when compared to the control group.
• There were no differences between ear sides considering tube dysfunction.
• Females had higher occurrences of tube dysfunction.
